# Perceptions of antenatal iron-folic acid supplements in urban and rural Pakistan: a qualitative study

**DOI:** 10.1186/1471-2393-14-344

**Published:** 2014-10-01

**Authors:** Yasir Bin Nisar, Ashraful Alam, Brekhna Aurangzeb, Michael J Dibley

**Affiliations:** Sydney School of Public Health, The University of Sydney, Sydney, NSW 2006 Australia; Children’s Hospital, Pakistan Institute of Medical Sciences, Islamabad, Pakistan

**Keywords:** IFA supplementation, Perceptions, Pregnant women, Healthcare providers, Barriers

## Abstract

**Background:**

In Pakistan, 51% of women are anaemic in pregnancy yet only 44% of women use antenatal iron-folic acid (IFA) supplements. Little information exits on the perception and barriers to the use of IFA supplements during pregnancy in Pakistan. The aim of the study was to understand women and healthcare providers’ perceptions, and to investigate the cultural and behavioural factors influencing the use of antenatal IFA supplements in rural and urban settings of Pakistan.

**Methods:**

We conducted 10 focus group discussions with mothers, 10 in-depth interviews with currently pregnant women, 6 in-depth interviews with Lady Health Workers and 4 in-depth interviews with doctors providing antenatal care services. The study was conducted in two districts of Pakistan - district Swabi and Islamabad for rural and urban samples, respectively. Data was collected between August and November 2012.

**Results:**

The majority of women were aware of the perceived benefits of antenatal IFA supplements. However, the rural women had more limited information about the benefits of IFA supplements than the urban women. The facilitating factors for the women’s use of supplements were: they had knowledge of benefits; they had trust in the healthcare providers; the supplements were available; they had the financial capacity to buy them; they felt better after taking these supplements; and they received support from family members. The barriers to the women’s use of supplements were: they forgot to take them; the non-availability of supplements; their limited financial capacity to buy them; the lack of antenatal care services; family members not allowing use of the supplements; not knowing about the benefits or no education; fear or experience of side effects; considering them as contraceptives; and felt better thus stopped.

**Conclusion:**

The coverage of antenatal IFA supplementation can be improved by reducing the barriers related to the use of antenatal IFA supplementation in Pakistan. Interventions focused on providing adequate awareness, good quality counselling, reminder messages, availability of free supplements throughout pregnancy and reducing the side effects should be developed and implemented.

## Background

There are 1.62 billion anaemic people, which is a quarter of the global population [1]. Approximately 42% of pregnant women are anaemic worldwide [1], whereas the prevalence of anaemia among Pakistani pregnant women is higher with 51% of them anaemic at any stage during their pregnancy [2]. Iron deficiency is the most common cause of the anaemia during pregnancy and it is the most widespread nutritional deficiency in the world [3, 4]. It has been observed that anaemia during pregnancy is associated with an increased risk of maternal mortality [5], low birth weight [6–8] and prematurity [6, 8, 9]. To prevent anaemia during pregnancy, the World Health Organization recommends, as a part of antenatal care (ANC) programs, a standard daily oral dose of 30–60 mg iron and 400 μg folic acid supplements to begin as early as possible and continue throughout pregnancy [10]. A recently published meta-analysis has identified a significant reduction of 19% in the risk of low birth weight associated with the use of antenatal iron supplements [11]. Further, a pooled analysis of data from four Indonesian demographic and health surveys (1994, 1997, 2002–03 and 2007) showed a significant 39% reduction in neonatal mortality with antenatal iron-folic acid (IFA) supplementation [12].

In Pakistan, antenatal IFA supplements (elemental iron 60 mg and folic acid 0.5 mg) are distributed free of cost by maternal and child health services through the existing primary healthcare system including health facilities and the community health worker program, known as the Lady Health Workers (LHW) program for daily consumption throughout the pregnancy starting from second trimester. However, the overall level of consumption of antenatal IFA supplements at any stage during pregnancy is currently low in Pakistan (44%), and is even lower for rural women (38%) [13]. Moreover, 16% overall and 12% of the rural women consumed 90 or more supplements throughout their pregnancy [13]. In comparison with other South Asian countries such as Bangladesh [14], India [15], and Nepal [16, 17] the coverage of antenatal IFA supplements is the lowest in Pakistan [13]. The current low coverage of IFA supplements needs to be investigated further to understand the issues related to these programs in Pakistan. Further research in this field will help to develop interventions to improve the coverage of antenatal IFA supplementation.

Evidence from developing countries suggests that the outcome of antenatal IFA supplementation programs is influenced by the behaviour of the pregnant women and the healthcare providers. The pregnant women reported they forget or were unwilling to take the supplements, while healthcare providers gave inadequate counselling and distribution of iron tablets [18]. Qualitative research can help in understanding the behaviour of the pregnant women and their healthcare providers related to the use of IFA supplements. Subsequently, the findings of this qualitative research can assist stakeholders from the government and non-government organizations (NGOs) to develop and implement better behaviour change communication interventions to improve the coverage in Pakistan. The aim of the current study was to understand the women and the healthcare providers’ perceptions and to investigate the cultural and behavioural issues related to the use of antenatal IFA supplements in rural and urban settings of Pakistan.

## Methods

### Study design, study sites and population

In this study, in-depth interview (IDI) and focus group discussion (FGD) methods were used to capture data from selected rural and urban settings in two districts of Pakistan. The rural sample was selected from the district of Swabi while the urban part was conducted at the Pakistan Institute of Medical Sciences (PIMS), Islamabad. PIMS is the largest tertiary care, teaching hospital in Islamabad, the capital city of Pakistan. The institute has three major components – the Islamabad Hospital for adult patients with many sub specialties; the Children’s Hospital for paediatric patients; and the Maternal and Child Healthcare Centre for gynaecology and obstetrics patients.

The two study districts were selected based on predominant urban and rural settings, socioeconomic status and prevalence of use of IFA supplements during pregnancy. In Swabi district, 69% of women had no education, the total fertility rate was 3.1, 67% of respondents had at least one ANC visit [19] and 45% of women consumed antenatal IFA supplements during their most recent live birth 5 years prior to the survey [20]. In comparison, in Islamabad region 16% of women had no education, the total fertility rate was 3.0, 97% of women had at least one ANC visit and 80% of women consumed antenatal IFA supplements during their recent live birth within 5 years prior to the survey [21].

Four main categories of participants were identified who are involved in the antenatal IFA supplementation and therefore, we included participants from these four categories in the study. These are: (a) the rural and the urban currently non-pregnant mothers who gave birth within five years prior to the study; (b) the rural and the urban currently pregnant women; (c) the community health workers [Lady Health Workers (LHWs)] who are responsible for providing antenatal IFA supplements to pregnant women in the rural communities; and (d) the doctors working in a tertiary care hospital, who are providing IFA supplements to pregnant women in the urban settings. We did not consider including traditional birth attendants because antenatal care services are mainly provided by healthcare providers and only 2.4% of rural women received ANC services from traditional birth attendants in Pakistan [21]. The details of background characteristics of participants are given in Table 1.

**Table 1:** **Basic characteristics of respondents**

Characteristics	Currently pregnant women^1^(n = 10)		Currently non-pregnant women^2^(n = 73)	
	Rural (n = 6)	Urban (n = 4)	Rural (n = 47)	Urban (n = 26)
**Age**				
Less than 25	3 (50.0%)	2 (50.0%)	8 (17.0%)	6 (23.1%)
25 to 34 years	1 (16.7%)	1 (25.0%)	19 (40.4%)	12 (46.2%)
35 and more	2 (33.3%)	1 (25.0%)	20 (42.6%)	8 (38.7%)
**Respondents educational status**				
Illiterate	3 (50.0%)	1 (25.0%)	29 (61.7%)	5 (19.2%)
Primary	2 (33.3%)	1 (25.0%)	12 (25.5%)	6 (23.1%)
Secondary and above	1 (16.7%)	2 (50.0%)	6 (12.8%)	15 (57.7%)
**Husband's educational status**				
Illiterate	2 (33.3%)	1 (25.0%)	22 (46.8%)	3 (11.5%)
Primary	2 (33.3%)	0 (0.0%)	16 (34.0%)	7 (26.9%)
Secondary and above	2 (33.3%)	3 (75.0%)	9 (19.1%)	16 (61.5%)
**Duration of current pregnancy**				
First trimester	1 (16.7%)	1 (25.0%)	NA	NA
Second trimester	1 (16.7%)	3 (75.0%)		
Third trimester	4 (66.6%)	0 (0.0%)		
**Number of children**				
First time pregnant	1 (16.7%)	2 (50.0%)	NA	NA
1 to 3	3 (50.0%)	2 (50.0%)	26 (55.3%)	16 (61.5%)
4 and more	2 (33.3%)	0 (0.0%)	21 (44.7%)	10 (38.5%)
**Age of the youngest child**				
First time pregnant	1 (16.7%)	2 (50.0%)	NA	NA
Less than 18 months	4 (66.6%)	0 (0.0%)	14 (29.8%)	13 (50.0%)
18 to 36 months	1 (16.7%)	2 (50.0%)	21 (44.7%)	6 (23.1%)
More than 36 months	0 (0.0%)	0 (0.0%)	12 (25.5%)	7 (26.9%)

^1^Currently pregnant women were involved in in-depth interviews.

^2^Currently non-pregnant women were involved in focus group discussion.

### Ethics

Informed consent was obtained from each of the participants for the IDIs and FGDs. Ethical and institutional approvals were obtained from the Hospital Ethics Committee, Pakistan Institute of Medical Sciences, Islamabad, from the Lady Health Workers’ program, Swabi district, and from the Human Research Ethics Committee, the University of Sydney, Australia.

### Sampling

**Figure 1:**
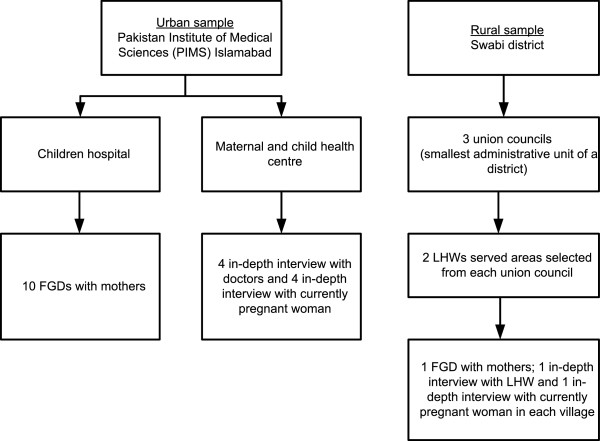
**Sampling frame for the study.**

Sampling was purposive and the respondents were selected from the urban and the rural areas. Figure 1 summarizes the sampling frame used in the study. The smallest administrative unit in a district is a union council, with an average population of 10,000 people and has a government primary health facility (basic health unit/ rural health centre). In Swabi district, there are 56 union councils. We selected three primarily rural union councils, with operational government primary health facilities and served by the LHW program. From each union council, two LHW served areas were selected. In each LHW area, one FGD with mothers and one IDI each with pregnant woman and LHW were carried out. In the urban setting, both FGDs and IDIs were carried out at the PIMS, Islamabad. The doctor on duty to providing ANC services at the outpatient department of the PIMS Maternal and Child Health Centre at the time of data collection was interviewed. The currently pregnant women who visited the duty doctor at the PIMS Maternal and Child Healthcare Centre, for ANC services on that day were also interviewed. The currently non-pregnant mothers from Islamabad, who came with their sick children to the Children’s Hospital of the PIMS were invited for FGDs.

### Data collection process

Separate guidelines were developed for the FGD with the currently non-pregnant women/mothers and for the IDIs with the currently pregnant women, LHWs and the doctors. These interview tools were translated into Urdu (the language spoken by the study participants). A female doctor and two social scientists were hired and trained in the study objectives, methodology and the guidelines. We pre-tested the data collection guidelines in the urban and the rural communities of the participating districts, but in locations where actual data collection was not done. The guidelines were then modified on the basis of the findings of the pre-testing. The major topics contain perceptions about iron-folic acid supplements, its benefits/disadvantages, and barriers and facilitating factors for IFA supplements use in pregnancy.

In rural areas, the village leader of the selected LHW area was informed about the purpose of the study before the commencement of data collection. The village leader contacted the LHW working in the selected area and the investigators invited her to participate in the study. Then, the local LHW identified the currently non-pregnant mothers and the pregnant women living in her village and the investigators invited them to participate in the study. Moreover, the women for IDI were further stratified by socio-economic status, with participants from three different classes, such as high socio-economic status, middle socio-economic status and low socio-economic status.

After informed consent, the session/interview was commenced according to the guidelines. A series of questions were asked to understand the perceptions of the participants about the use of antenatal IFA supplementation. Furthermore, the behavioural and cultural issues in terms of the facilitating factors and the barriers to the use of antenatal IFA supplementation were also explored. The average time for the FGDs was 1 hour and 20 minutes and 45 minutes for the IDI. All the information/discussions were audio recorded and the key information was also noted by hand. At the end of each session, the investigators provided, to all the participants, information about the use of antenatal IFA supplementation, and its impact on maternal health, and the growth and development of the newborn. The average number of participants per FGD was 7 women.

### Data analysis

Two investigators (YBN and BA) independently transcribed the audio recorded interviews and discussions verbatim and expanded the field notes in Urdu (the language spoken by the study participants). Audio-recordings and notes were then translated into English. YBN and BA developed a list of topical codes for all topics related to the research questions that was independently reviewed and verified by another investigator (AA). Subsequently the text pertaining to each topical code was discussed and summarised in a document that presents the findings for each topic using quotes and tables. NVivo version 10 (QSR International, Victoria Australia) was used to aid the data organisation and analyses.

## Results

### Profile of study participants

Forty seven rural and 26 urban mothers participated in 6 and 4 FGDs respectively. The IDIs were conducted with six rural and 4 urban currently pregnant women. The basic characteristics of all women, who participated in the study from the rural and urban communities, are shown in Table 1. The IDIs were conducted with the six LHWs working in the Swabi district and with the four doctors working at the PIMS Maternal and Child Health Centre, Islamabad.

### Perception of IFA supplements

The majority of the participating women were aware of antenatal IFA supplementation. The rural women knew the supplements by names such as ‘tablets to provide strength’ or ‘red tablets’. The rural women had limited information about the IFA supplements and their benefits. A few of the rural women reported about the supplements as providing strength to their weak bodies during the pregnancy; curing dizziness, lethargy and the back pain; improving maternal health and wellbeing; needing to be taken once daily and continued throughout the pregnancy; and to prevent complications during the pregnancy and at the time of delivery.

*“These tablets are good to provide strength to our bodies which are weak during the pregnancy, and also improve the feeling of dizziness; these tablets are good for my health”****(A rural mother of three children who participated in FGD).***

*“It prevents the complications during the pregnancy and at the time of delivery”****(A currently pregnant rural woman who participated in IDI).***

The urban women, on the other hand, were aware of the various brand names of the supplements available in the market. A substantial majority of the urban women were able to describe the supplements and reported the benefits of the supplements as improving the haemoglobin level; improving the appetite; curing anaemia; needing to be taken once daily and continued throughout pregnancy; improving maternal health and the health and growth of the foetus/baby; preventing delivery complications; and taking a good diet (such as vegetables and meat) along with the supplements.

*“These [supplements] are good to take during the pregnancy because these improve the haemoglobin levels and improve the health of mother and health and growth of the baby inside the mother’s womb”****(A currently pregnant urban woman who participated in an IDI).***

Nevertheless, some of the selected women were not aware of the antenatal IFA supplements. They had no information about the supplementation program and the benefits of the supplements. They reported several reasons for their lack of awareness and the common reasons included: no visits by the LHW because they lived some distance away; no visits by them to any health facility during pregnancy; poverty and no education; and no information given to them by the doctors even when they visited a health facility.

*“We live on the other side of the village and she [LHW] doesn’t come to our house; we are very poor and it is difficult for us to go to a hospital”****(A pregnant rural woman who participated in an IDI).***

All the participating LHWs and the doctors were aware of the IFA supplements and their health benefits. LHWs reported that the women in their communities knew about the supplements and called these supplements as ‘tablets to provide the strength’, ‘red tablets’ or ‘blood producing tablets’.

*“These tablets are good for the health of pregnant women because they face many hardships during their pregnancy; they feel weakness and dizziness; these tablets cure them and provide them strength; here [village] women know about these tablets as to provide strength, while some of them call them as tablets to produce blood”****(A LHW working for the last 7 years who participated in an IDI).***

The doctors reported that the majority of the pregnant women who visited the health facility knew about the supplements and that a few of them even knew the brand names available in the market.

### Source of information about antenatal IFA supplements

The great majority of the rural women reported receiving information about IFA supplementation from the LHWs, and a few of them also reported receiving information from doctors when they visited health facilities. The urban women received the information about the supplementation from a variety of sources. Most of them received information from doctors, but some of them also received information from their family members, friends, neighbours, televisions or from newspapers/magazines.

### Sources of antenatal IFA supplements

The major sources of the antenatal IFA supplements stated by the women were LHWs (rural women); government health facilities; and private clinics or pharmacies. The majority of rural women reported that when they visited healthcare providers for problems related to their pregnancy they received or were prescribed IFA supplements. The common problems for which they sought care included back pain, feeling weak, dizzy and lethargy. A few of them also visited the healthcare providers for routine ANC services and also received IFA supplements.

*“I was not feeling well few months ago, as I had severe back pain and feeling weak; my mother took me to a private clinic and the doctor over there prescribed me these tablets along with some other medicines”****(A rural woman pregnant for the first time who participated in an IDI).***

Among the urban women, some of them went to the health facility for the routine ANC services and received or were prescribed supplements; while others visited a health facility due to some problems related to their pregnancy and received the supplements. Occasionally, a family member also advised urban pregnant women to use these supplements.

*“I was admitted to the hospital because I had some internal problems with bleeding during my previous pregnancy; the senior doctor prescribed me these tablets at the time of discharge”****(An urban mother of three children who participated in a FGD).***

### Reasons to use antenatal IFA supplements - facilitating factors

The women reported several reasons for using IFA supplements during pregnancy including: they had knowledge of the benefits of the supplements; they had trust in the healthcare providers; the supplements were available and they had the financial capacity to buy them; they felt better after taking the supplements; and they received support from family members to take the supplements.

*“I know these tablets are good to provide strength to body which is weak during pregnancy, and also improve dizziness; these tablets are good for my health”****(A rural mother of three children who participated in a FGD).***

*“When I was pregnant my sister-in-law advised me to take these tablets because these tablets have advantages for myself and for the health of my child; afterward, doctor in this hospital also told me about them”****(An urban woman pregnant for the first time who participated in an IDI).***

LHWs reported that they provided the supplements to all the pregnant women because they were a part of the routine care during the pregnancy; to improve the health of the pregnant women; and to cure anaemia or low haemoglobin levels during the pregnancy.

*“When I visit them* [pregnant women] *for check up during their pregnancy, I provide them these tablets; sometime pregnant women have some health problems like weakness, dizziness, so I provide them these tablets and they feel better afterwards”****(A LHW working for 4 years who participated in an IDI).***

The doctors also stated various reasons to prescribe the supplements to all the pregnant women visiting them, including that they are a part of routine ANC services; due to poor dietary habits of the pregnant women; due to signs or symptoms of anaemia, or low measured haemoglobin levels; and to improve maternal health and growth and the development of the newborn.

### Timing of the start of antenatal IFA supplements

A substantial majority of the women started the antenatal IFA supplementation during the second trimester of their pregnancy. LHWs reported distributing the IFA supplements to all the pregnant women in their catchment area from the 4^th^ or 5^th^ month of their pregnancy onwards. While doctors also reported that they prescribed IFA supplements during the second trimester of pregnancy to the pregnant women who visited the hospital, either for routine check-ups or with some specific problems.

### Barriers to antenatal IFA supplementation

The common barriers to the use of IFA supplements reported by the urban and rural women included forgetting to take them on a daily basis; the non-availability of the supplements; their limited financial capacity to buy them; no ANC services available; family members such as mother-in-law, husband or mother did not allow them to use the supplements; they did not know about the benefits and they had no education; they were afraid of the supplements or they had experienced side effects (like vomiting, nausea or constipation); and they considered them to be like contraceptives. In addition, the urban women also reported that they felt better and stopped the IFA supplementation after few days without consultation with their healthcare providers.

*“I stopped these after few days because I forgot to take on each day as I have many duties to perform at home and difficult to remember on each day”****(A rural mother of three children who participated in a FGD).***

*“I used these tablets but after few days I had vomiting and diarrhoea with these and my mother-in-law told me to stop this medicine; she [mother-in-law] told me not to take any medicine during pregnancy”****(An urban currently pregnant woman who participated in an IDI).***

According to healthcare providers the common barriers to the use of IFA supplements were: the women forgot to take them daily; family members mainly mother-in-law and husband did not allow the women to use them; the woman felt better and stopped without consultation; the women did not take medicines during the pregnancy; the supplements were not available or the woman had limited financial capacity to buy them; the woman did not visit healthcare providers; fear of the supplements or previous experience of the side effects of the supplements; the women did not know the advantages of these supplements; and the women had no education.

*“There are some women in my village who do not follow our advice because their family [husbands or mothers-in-law] don’t allow them; as they think it is bad to take medicines during pregnancy because medicines have some negative effects on baby’s health”****(A LHW working for 12 years who participated in an IDI).***

The majority of the women were of the view that they would like to use the supplements in the future. However, some of them wanted to receive the supplements in an adequate quantity and free of charge throughout the pregnancy. They also wanted to recommend the supplements to other pregnant women in their family or neighbourhood. A few of them, who had experienced some side effects or were not aware of the benefits associated with the supplements, did not want to recommend the supplements to other pregnant women.

### Pathways of procurement of antenatal IFA supplements by a pregnant woman

**Figure 2:**
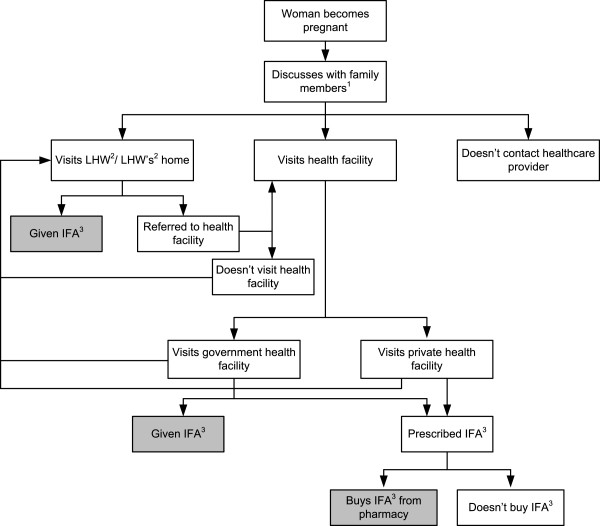
**Pathways for the procurement of IFA supplements by a pregnant woman.** ^1^ Family member: mother-in-law, mother, husband or sister-in-law. ^2^ Lady Health Worker. ^3^ Iron-folic acid supplements.

Figure 2 summarizes the women’s and the healthcare providers’ perceptions of the process of procurement of antenatal IFA supplements by a pregnant woman. When a woman becomes pregnant, she is likely to discuss her pregnancy and what she needs to do with her family members, such as mother-in-law, husband, mother or sisters-in-law. Often, a family member will take her to the health facility (government/private) or LHW (in the rural communities), for routine check-ups or for a health problem. If the woman visits a LHW, the IFA supplements when available are provided free of charge, but if the LHW does not have a supply, the woman will be referred to a government health facility. If she visits a government health facility, she will either receive the IFA supplements free of charge, or if the supplements are not available at the government health facility, the doctor will prescribe them for her. Similarly, if she visits a private clinic, the healthcare provider prescribes her the supplements. Once she gets the prescription, sometimes she will purchase the supplements from a pharmacy. A pregnant woman will not use IFA supplements during her pregnancy if she does not receive any ANC services, or if her family members (mother-in-law, husband, mother) do not allow her to use them, or if she avoids taking medicines during pregnancy, or if she is not aware of IFA supplements and their benefits, or if she has limited financial capacity to purchase the supplements.

### Pathways of consumption of antenatal IFA supplements by a pregnant woman

**Figure 3:**
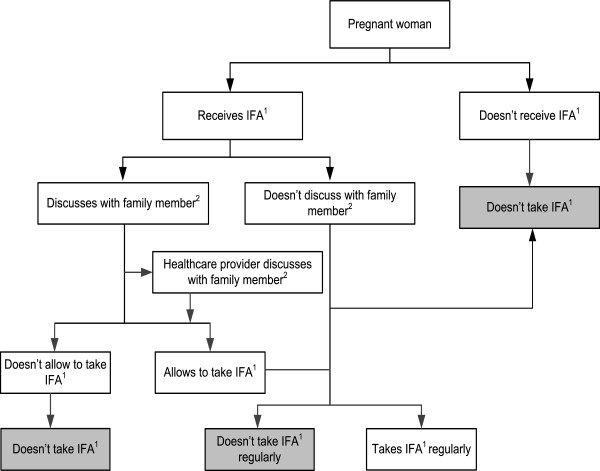
**Pathways for the consumption of IFA supplements by a pregnant woman.** ^1^ Iron-folic acid supplements**.** ^2^ Family member: mother-in-law, mother, husband or sister-in-law.

Figure 3 summarizes the pathways of perceptions of the women and the healthcare providers about the consumption of IFA supplements by a pregnant woman. Once a pregnant woman receives the supplements, sometimes she discusses their use with a family member mainly with her mother-in-law, husband, and mother or occasionally with her sisters-in-law, who may or may not allow her to take them. She may continue taking the supplements regularly if she perceives the benefits of the supplements, or if she trusts her healthcare provider, or if the supplements are available, or if she has the financial capacity to purchase them, or if her family members support her taking them, or if she feels an improvement in her health. She may not take IFA supplements at all or she may stop the supplements after using them for few days or weeks if she forgets to take them each day, or if she is not aware of the need to continue taking them throughout her pregnancy, or if she feels better and stops without consulting her healthcare provider, or if she is not aware of the advantages of the supplements, or if she is not able to obtain a continuing supply of the supplements (non-availability), or if she has limited financial capacity to buy them, or if she experiences some side effects.

## Discussion

### Main findings and their significance

This study found that most of the women were aware of antenatal IFA supplementation and the benefits of the use of these supplements during pregnancy for maternal health and growth of the foetus/baby. However, the rural women’s perceptions about the benefits of the use of antenatal IFA supplements were mainly focused on maternal health. Whereas, the urban women had better knowledge and they were more aware of the advantages of supplementation such as curing anaemia, improving haemoglobin levels and improving foetal growth and development. The healthcare providers reported prescribing or providing the supplements to all the pregnant women who visited them. The information received by the pregnant women about the supplements from the healthcare providers was not uniform and did not explain the various aspects of the supplements, such as the description and management of minor side effects, possible ways to remember to take the supplements daily, and explanations of the advantages of the supplementation. The current study also investigated several facilitating factors and barriers to the use of IFA supplements. The recognized behavioural and cultural issues which influence the use of IFA supplements among urban and rural Pakistani women can play a useful role in the development of interventions to improve the coverage. There is a need to develop behaviour change communication interventions for IFA supplementation at the national, provincial and the district level. Further, a separate set of interventions are required to test in urban and rural settings of Pakistan to improve the coverage of IFA supplements.

### Strengths and limitations

The current study provides valuable information about the perceptions of women and the healthcare providers about the antenatal IFA supplementation in the urban and the rural communities of Pakistan. However, the results are not intended to be representative of all districts in Pakistan. The major strength of our study is that we conducted interviews with mothers, currently pregnant women and the healthcare providers including the community health workers and the doctors providing ANC services using different qualitative data collection techniques. Further, the sample was selected from urban and rural communities to understand and compare the perceptions of women and healthcare providers in these settings.

There are several limitations of this study. We did not collect data about perceptions of family members such as husband, mother-in-law and mother, and the health managers in the department of health about the antenatal IFA supplementation. Women who usually come to PIMS for ANC services may have better knowledge of IFA supplements, which might be a study limitation. However, we wanted to investigate the perceptions of women, who usually come to a health facility for ANC services and thus included the women receiving ANC services from the PIMS as well. We did not investigate the quality of counselling and information imparted by the healthcare providers by direct observation at the time of provision/ prescription of the IFA supplements. Hence, further investigation is needed to examine these issues.

### Facilitating factors

Facilitating factors for the use of antenatal IFA supplements recognised in our study were the perceived benefits of supplementation; the trust in the healthcare providers; the accessibility of the supplements and the financial capacity to buy them; support for their use from family members; and the experience of positive impact of the supplements.

Our study findings are consistent with other studies. Perceived and experienced benefits of the IFA supplementation was one of the important facilitating factors in our sample. Studies from the developed countries like the USA [22] and Sweden [23] have reported perceived and experienced benefits of the supplementation as important motivating factors for the use of supplements during pregnancy. Similarly, studies from the developing countries such as Senegal [24], Nigeria [25], and India [26] found this as a key factor for the consumption of IFA supplements during pregnancy. A multi-country study including eight countries from Asia, Latin America and Africa also reported perception or experience of benefits was an important facilitating factor [18]. Research has shown that pregnant women complied with supplementation when they were provided the necessary information at the time IFA supplements were provided [27]. It is, therefore, important for healthcare providers to explain all the advantages of IFA supplementation to the pregnant woman at the time the supplements are provided or prescribed*.* Enhanced training of healthcare providers and use of mobile technological can improve the counselling skills of healthcare providers especially in rural areas.

Our study showed that women used IFA supplementation if they had confidence and trust in the healthcare providers who prescribed or provided the IFA supplements. It is important that healthcare providers develop strong interactions with pregnant women while providing them with ANC services. Interaction and confidence in healthcare providers has been recognized as an important factor for the use of antenatal IFA supplements [22–25]. Good counselling from a healthcare provider has been reported to improve the compliance with IFA supplementation during pregnancy [27].

Accessibility or financial capacity to buy the supplements was also a facilitating factor in our study. Research has shown that easy accessibility, supplements free of charge or even the availability of low cost supplements improved the use of antenatal IFA supplements [22, 24, 27]. In Pakistan, free IFA supplements are being distributed to pregnant women through the government health facilities and the LHW program. Women who live close to the government health facilities or in a LHW program area can obtain IFA supplements free of charge. However, the supplements are often neither available with LHW nor at government health facilities. There is a need to strengthen the current logistic system of LHW program and the health department at district level. Whereas, sometimes it is difficult for women who either do not live in a LHW service area, or are far from a government health facility to obtain these supplements free of charge. Alternate ways such as involvement of community volunteers and traditional birth attendants through non-governmental organizations for provision of the supplements to the pregnant women in non-LHW service areas should also be explored.

An important facilitating factor for IFA supplementation in our sample was the support from the family member, which has also been identified by other investigators [22, 25, 26]. Pakistani women during their pregnancies usually dependent on the decisions of their family members (particularly husband, mother-in-law or mother) on several occasions including utilizing ANC services, taking medicines, deciding the place of delivery, type of delivery assistance and the need for postnatal care. While, most of the time, the decisions of family members depend on their financial capacity and their level of education. There is a need to provide health education to family members such as husband, mother-in-law and mother through community level advocacy campaigns.

### Barriers

Similar to many other studies forgetting to take supplements daily was the most common barrier to antenatal IFA supplementation in the women we studied [18, 22–24, 28]. Women have reduced short-term memory during pregnancy and a recently published systematic review has found that pregnant women performed more poorly than non-pregnant women on memory and other cognitive tests [29]. At the same time, many pregnant women have to perform several tasks at home throughout the day and they often forget to take the supplements on a daily basis. As suggested by one of the doctors in our study, ‘pregnant woman should keep the supplements in home where she spends most of the time during the day like in kitchen’. Further, there is a need to provide continuous messages to remind the pregnant women through family members, healthcare providers or through the use of mobile phones to improve compliance with the daily consumption of antenatal IFA supplements [30].

Non-availability or limited financial support to purchase the supplements after receiving prescriptions from healthcare providers was one of the barriers to the use of antenatal IFA supplements in the women we interviewed. Others have also reported similar findings in their studies [18, 24, 31]. Distribution of IFA supplements through community health workers is a good alternative to distribution by health facilities in developing countries, where accessibility to health facilities is limited [18, 32]. However, often it is difficult for the pregnant women to receive adequate quantity of supplements due to frequent stock out and the provision of supplements on a quota system (not according to actual demand) to the LHWs. There is a need to strengthen the logistic system for supplying IFA supplements to LHWs as well as to the health departments at the district and the provincial levels.

Many women stated that the experience or fear of gastrointestinal side effects as one of the barriers to the use of IFA supplementation during pregnancy. Several studies have considered the presence of side effects as the major barrier to the IFA supplementation [18, 22–24, 26, 28, 31], leading many to advocate weekly instead of daily supplementation, and others to suggest alternative iron preparations to reduce side effects [33]. However, a study from Bangladesh found that the side effects have limited impact on the use of IFA supplements during pregnancy [34]. Women, even having some minor gastrointestinal side effects, continued using the supplements if they perceived or experienced benefits of the supplementation [18, 26]. Hence, the quality of counselling is important to overcome this problem.

As with other studies [18, 24, 31], we found the lack of awareness of the benefits of IFA supplements, lack of knowledge to continue the supplements or feeling better and stopping the supplementation were also considered as barriers to the use of IFA supplements. A clear message at the time of initiation of the supplements by the healthcare provider can overcome this barrier. Similarly, alternative counselling techniques like use of mobile phones messages [30] and counselling in places of worships such as mosque have shown a positive impact on the use of IFA supplements in Nigeria [25].

### Program implications

Our study findings have implications for the IFA supplement distribution programs in Pakistan. In low- and middle-income countries where it is often difficult to reach health facilities in resource poor communities, the distribution of the IFA supplements through community health workers is a successful approach [18, 32]. In this context, the LHW program, which was launched in 1994 by the Government of Pakistan, has been providing ANC services including supply of IFA supplements to all the pregnant women in its catchment area [35]. Currently, about 60% of the total population of Pakistan is covered by the LHW program. Based on our findings, to improve IFA supplementation in the LHW service areas there is a need to retrain the LHWs for better counselling, reminder text and voice messages by using the mobile technology [30], and to improve the logistic system of the program on current demand and supply. At the same time, in the non-LHW program areas, where the government health facilities are the only source for free supplementation, alternative ways to reach the pregnant women like recruiting volunteers and involving traditional birth attendants in the distribution program of the supplements should also be explored. Nepal has improved the IFA supplementation coverage from 23% in 2001 [36] to 80% in 2011 [16] by running a package of successful interventions [37]. There is a need to modify and test the feasibility and effectiveness of these interventions through multi-sites, community based trials with large sample size in resource poor communities of Pakistan.

## Conclusions

The current study reveals that awareness of benefits, confidence in healthcare providers, accessibility or financial capacity, receiving support from family members and experience of the health benefits of the supplements were the facilitating factors for the use of antenatal IFA supplements in rural and urban settings of Pakistan. Forgetfulness, the non-availability or lack of financial capacity to buy, experience of gastrointestinal side effects, lack of knowledge about the benefits, and the lack of knowledge to continue, or stopping on recovery were the barriers to the use of antenatal IFA supplements in rural and urban areas of Pakistan. The cultural and behavioural factors recognized as the facilitating and barriers to the use of IFA supplements are important for the development of interventions. There is a need to develop and apply behaviour change communication interventions, possibly using mobile technologies, to improve the coverage of antenatal IFA supplementation.

## Abbreviations

ANC: Antenatal care
FGD: Focus group discussion
IDI: In-depth interview
IFA: Iron-folic acid
LHW: Lady health workers
NGOs: Non-government organizations
PIMS: Pakistan institute of medical sciences.
